# Homeless Aging Veterans in Transition: A Life-Span Perspective

**DOI:** 10.1155/2013/570407

**Published:** 2013-10-27

**Authors:** Carla J. Thompson, Nancy L. Bridier

**Affiliations:** ^1^University of West Florida, Pensacola, FL 32514, USA; ^2^Research and Advanced Studies, University of West Florida, 11000 University Parkway, Building 78, Office 121, Pensacola, FL 32514, USA

## Abstract

The need for counseling and career/educational services for homeless veterans has captured political and economic venues for more than 25 years. Veterans are three times more likely to become homeless than the general population if veterans live in poverty or are minority veterans. This mixed methods study emphasized a life-span perspective approach for exploring factors influencing normative aging and life-quality of 39 homeless veterans in Alabama and Florida. Seven descriptive quantitative and qualitative research questions framed the investigation. Study participants completed a quantitative survey reflecting their preferences and needs with a subset of the sample (*N* = 12) also participating in individual qualitative interview sessions. Thirty-two service providers and stakeholders completed quantitative surveys. Empirical and qualitative data with appropriate triangulation procedures provided interpretive information relative to a life-span development perspective. Study findings provide evidence of the need for future research efforts to address strategies that focus on the health and economic challenges of veterans before they are threatened with the possibility of homelessness. Implications of the study findings provide important information associated with the premise that human development occurs throughout life with specific characteristics influencing the individual's passage. Implications for aging/homelessness research are grounded in late-life transitioning and human development intervention considerations.

## 1. Introduction

The special population of homeless veterans has become an alarming concern within America's aging population challenges. According to the National Coalition for Homeless Veterans [1], while only 7% of the US population is comprised of veterans, approximately 13% of the adult homeless populations are veterans. Recent national survey efforts conducted by the Veterans' Administration and related government agencies report that 8.6% of homeless veterans are over the age of 62 with aging female homeless veteran populations increasing each year [2]. Veterans are three times more likely to become homeless than the general population if they live in poverty or are minority veterans in poverty. “Low income veterans are twice as likely to become homeless than the general population of low-income adults” [1, paragraph 2]. Almost half of homeless veterans in the United States are over the age of 51 and are comprised primarily of veterans representing the Baby Boomers and are veterans of the Vietnam War years [3]. This mixed methods study emphasized a life-span development perspective modeled by [4] for addressing the issue of aging homeless veterans. The study explored expressed needs and commentaries of 39 homeless veterans within two states in the southeast region of the United States (Alabama and Florida). Seven research questions provided the focus areas of exploration. Quantitative survey data retrieved from a sample of 39 homeless veterans, qualitative individual interview data from a subset of *N* = 12 of the homeless veteran participants, and additional quantitative survey data from *N* = 32 service providers and stakeholders were retrieved to represent multiple perspectives for conducting triangulation of data procedures and interpretation. Specific materials and methods, including the background and theoretical framing of the investigation, study results, implications, and conclusions comprise the discussion.

## 2. Materials and Methods

 Key preliminary tasks for determining the materials and methods used in the study included an overview examination of the background and the literature considerations pertinent to the investigation and the theoretical underpinnings for the study. These preliminary efforts provided the impetus for the research questions and hypotheses as well as the rationale for the selection of specific materials and methods for the study.

### 2.1. Background and Statement of the Problem

 The need for services for homeless veterans has captured political and economic venues for more than 25 years. Aging homeless veterans who are not identified as in need of services for substance abuse are not eligible for existing Veterans' Administration services that include safe housing or shelter services [5]. Often aging homeless veterans who are not classified with service-related medical or health issues are also not provided access to resources and needed services offered by the Veterans' Administration. According to the Department of Veterans Affairs [5], eligibility for the Housing and Urban Development-Veterans Affairs Supportive Housing (HUD-VASH) program requires recipients to participate in VA provided case management and clinical services. The term, homeless veteran, is often depicted as chronically homeless by The McKinney-Vento Homeless Assistance Act as amended by S. 896, The Homeless Emergency Assistance and Rapid Transition to Housing (HEARTH) Act of 2009 [5]. Chronically homeless refers to a homeless individual who meets three criteria:

*(i) homeless and lives or resides in a place not meant for human habitation, a safe haven, or in an emergency shelter; (ii) has been homeless and living or residing in a place not meant for human habitation, a safe haven, or in an emergency shelter continuously for at least 1 year or on at least 4 separate occasions in the last 3 years; and (iii) has an adult head of household (or a minor head of household if no adult is present in the household) with a diagnosable substance use disorder, serious mental illness, posttraumatic stress disorder, cognitive impairments resulting from a brain injury, or chronic physical illness or disability, including the cooccurrence of 2 or more of those conditions [5, page 4]. *

Recent efforts by the President Obama administration to prevent and end veteran homelessness by 2015 are in progress and are focused on the goal of “finding every veteran safe housing and access to needed services” [6, paragraph 6]. In addition, the VA has worked jointly with local communities since 1993 on the Community Homelessness Assessment, Local Education, and Networking Groups (CHALLENG) for Veterans project [7]. The primary mandate of the previous CHALENG studies has been to “bring together VA and community service providers to foster coordinated services for homeless veterans” [7, page 23]. However, there has been little effort by the government to explore characteristics of homeless aging veterans and their respective needs (and changing needs) for services associated with specific individual development needs, such as education, mental health assistance, and employment. Since 96 percent of homeless veterans are alone (individuals) as compared with 63 percent of the overall homeless population, the need for examining specific individual characteristics of homeless veterans is a necessary prerequisite for determining appropriate services for this special population [8]. Although combat may be one of the most stressful experiences a veteran faces, many veterans face homelessness for a period longer than their war zone service [9]. The Interagency Council on Homelessness [9] reports that the average length of homelessness for a veteran is six years compared with four years for a nonveteran. The number of years a veteran remains homeless is important because the longer a person stays on the streets, the greater the possibility of developing health problems including physical health illnesses, mental disorders, and substance abuse issues [10]. Although previous studies indicate combat exposure and posttraumatic stress disorder (PTSD) were not found to be significant predictors of veterans becoming homeless, the impact of PTSD and the associated literature is evolving and changing as new research findings are discovered [11]. 

The state of Florida is ranked third in the nation in 2011 for the largest number of aging homeless veterans (with California ranked as the greatest number of homeless veterans), yet the numbers of services and special needs provided for Florida aging homeless veterans is lower than many states that serve far fewer number of homeless aging veterans [1]. The Veterans' Administration (VA) provides “beds” and other related homeless services only for those homeless veterans who are identified as substance abusers or have medical problems. The literature review efforts of previous research studies aimed at homeless veterans within the Gulf Coast region and preliminary overview examinations of existing programs currently servicing homeless veterans within the Northwest Florida region produced very limited information, especially regarding specific target groups of aging homeless veterans and aging homeless female veterans. Currently, services for homeless veterans in the northwest Florida region are limited to services for VA eligible veterans and first response facilities. Since the Florida panhandle region hosts multiple military establishments and since large numbers of military retired persons live in the region, the numbers of homeless veterans within the region parallel this pattern. In addition, the need for determining specific empirical information regarding how aging affects the needs of homeless veterans, especially homeless veterans within northwest Florida is evidenced in the lack of statistical information currently available for this region. The current study focused on northwest Florida and the surrounding region (including Alabama) for accessing participants and agencies not serviced by the Veterans' Administration. Homeless veterans and associated stakeholders from this region comprised the participants for the study. The significance of the study for the northwest Florida region and for the field of research associated with aging homeless veterans is evidenced in the staggering numbers of aging homeless veterans in Florida (especially women veterans) and the lack of programs for assessment and service specifically located within the Florida panhandle region [7]. 

 The current study addressed the need to focus on the individual. The study investigated factors contributing to the expressed needs of homeless aging veterans (who are potentially transitioning from homeless to nonhomeless status) relative to specific individual characteristics from a life-span perspective. The individual's reactions and interactions with the environment relative to the various gains and losses experienced by the individual in life are specific to the types of characteristics and descriptive information indicative of a life-span perspective. This premise has underpinnings within the context of the life-span perspective of human development theory. 

### 2.2. Theoretical Framework

**Figure 1:**
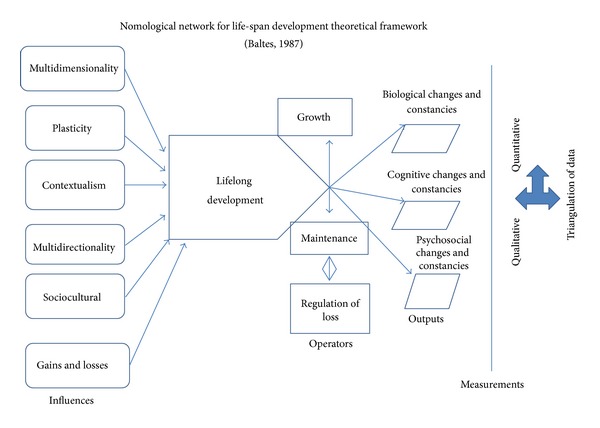
Nomological network for life-span development theoretical framework [4].

 The theoretical framework for the study focuses on the individual and is grounded within the context of the life-span perspective of human development theory posited by Baltes [4, 12, 13]. The life-span perspective of human development premise describes development as lifelong, sociocultural, multidimensional, multidirectional, plastic, contextual, and influenced by biology and culture involving a process consisting of growth, maintenance, and regulation of loss [4, 12, 14]. Sociocultural influences are inherent within the setting or environment in which the individual lives. Multidirectional influences include the positive and negative events experienced by the individual within the course of human development. Multidimensionality is the perspective that there are multiple pathways in an individual's development [15, 16]. Plasticity is the ability of the individual to adapt to life changes. Contextual influences include the types of changes in resources that occur in response to the individual's needs for resources throughout a changing life-span. Life-span development involves biological considerations, cultural considerations, and individual factors working together. The life-span perspective emphasizes development across the course of a lifetime, and all stages of the lifetime span contribute to development. The life-span theoretical framework, as displayed in Figure 1, established the foundation for the research questions, hypotheses, instrumentation, methodology, and triangulation of data alignment used in the study. 

### 2.3. Specific Purpose and Objectives of the Study

 The specific purpose of this mixed methods study was to explore the individual characteristics and needs of aging homeless veterans in transition within Alabama and northwest Florida relative to a life-span perspective. The study included four focused objectives: (1) to examine homeless veterans' perspectives utilizing a quantitative survey research approach and a qualitative interview inquiry research approach framed within the life-span development theory perspective of Baltes [4, 12]; (2) to conduct an intense focused interviewing effort of a subgroup of 12 aging homeless veterans within Alabama and northwest Florida, to determine specific characteristics and needs of this special population with an emphasis on individual considerations; (3) to conduct a quantitative survey research effort to obtain specific demographic and needs assessment information relative to the special population of aging homeless veterans and their respective service providers using the Veterans' Administration CHALLENG 2010 survey; and (4) to triangulate survey and interview data for the purpose of examining study results and emerging thematic findings relative to the constructs of life-span development theory.

### 2.4. Research Questions and Hypotheses

The theoretical framework assisted in guiding the development of the study within the context of examining the various constructs related to the individual's perspective of life-span development regarding specific gains, losses, and other influences as presented in Figure 1. Seven research questions (five quantitative research questions and two qualitative research questions) were generated from the theoretical framework development efforts.

*Research Question  1*. Is there a relationship between homeless veterans' levels of depression and the number of months they served in a war zone?

*Research Hypothesis  1*. There is a relationship between homeless veterans' levels of depression and the number of months they served in a war zone.

*Research Question  2*. Is there a relationship between whether or not a veteran served in a war zone and the homelessness status of the veteran?

*Research Hypothesis  2*. There is a relationship between service or no service in a war zone and whether or not the veteran becomes homeless.

*Research Question  3*. Is there a difference in the median rankings of perceptions of homeless veterans and service providers concerning VA accessibility?

*Research Hypothesis  3*. There is a difference between homeless veterans and service providers in their median rankings perceptions concerning VA accessibility.

*Research Question  4.* Is there a difference between the average perceptions of service providers in northwest Florida and service providers in Birmingham, Alabama, regarding their perceptions of VA community partnership integration?

*Research Hypothesis  4.* There is a difference between the average perceptions of service providers in northwest Florida and service providers in Birmingham, Alabama, regarding their perceptions of VA community partnership integration.

*Research Question  5.* How do the expressed needs of veterans in the current study differ from the expressed needs of veterans in the 2010 CHALENG studies?

*Research Hypothesis  5*. There is a difference in the rank ordering of the expressed needs of current study homeless veteran participants (from the Birmingham, Alabama, and northwest Florida regions) as compared with the CHALENG 2010 study participants.

*Research Question  6.* What are the major contributing factors to veteran homelessness, focused on life and/or military experiences that have impacted homeless veterans' development considerations leading to their current living situations?

*Research Question  7.* What are the immediate needs of homeless veterans?

### 2.5. Methodology

 The study utilized an exploratory mixed methods investigation focused on the characteristics and needs of the aging homeless military veteran population. The study involved two sequential procedural phases. The objective of the first phase was to determine the specific characteristics, perceptions, and needs of the homeless veteran participants. Phase one employed a survey instrument used to obtain demographic, perceptions, and needs information relative to aging homeless veterans who are currently not receiving housing services within the Birmingham, Alabama, and Northwest Florida regions. In addition, quantitative surveys were completed by service providers of homeless veterans. The second phase of the study consisted of qualitative interviews with a voluntary sample of aging, homeless veterans. Both quantitative and qualitative data were included in the data analysis procedures for the study. 

### 2.6. Study Participants

Study participants included 39 homeless veterans and 20 service providers from the Birmingham, Alabama, and Pensacola, Florida, areas. Homeless veterans were recruited to participate from the resident population of nonprofit organizations servicing homeless veterans within the two locations. A subset of 12 homeless veterans (of the 39 homeless veterans who participated in the quantitative survey) agreed to participate in the one hour qualitative interview session within a private room with an interviewer. The 20 service providers who agreed to participate in the study were comprised of employees of shelters, ministers, counselors, attorneys, and anyone who had consistent service experiences with homeless veterans. 

### 2.7. Instrumentation

 Both quantitative and qualitative instruments were obtained prior to the research effort. The quantitative survey instrument aligned with the national CHALENG 2010 study [7]. The qualitative instrument consisted of a structured interview protocol developed for the study. Instruments used in both phases of the research effort are described individually. 

#### 2.7.1. Quantitative Instrumentation

 The quantitative phase that employed the use of the Community Veteran Assessment instrument focused on the participant's demographic characteristics, length of time of homelessness status, length of military service/branch, psychological considerations, reported illnesses or mental health considerations, and expressed needs and services. The Community Veteran Assessment instrument was used in the Community Homelessness Assessment, Local Education, and Networking Groups (CHALLENG) for Veterans project [7] and utilizes a five-point Likert scale. Service providers completed an analogous instrument, also using the same five-point Likert scale to assess providers' characteristics, perceptions of VA accessibility and coordination, perceived value of the strategies most commonly used for collaboration between the provider and the VA, and the provider's perception of the needs of veterans. Reliability for the quantitative instrument used in the study was obtained by computing a Cronbach Alpha measure of internal consistency for each of the subscales inherent in the instrument. The resulting internal consistency alpha values for each of the Community Veteran Assessment subscales include Veterans' Needs Subscale (.98), Provider Strategies Subscale (.85), VA-Community Partnership Subscale (.86), and Veterans' Depression Levels Subscale (.46). 

#### 2.7.2. Qualitative Instrumentation

A semistructured interview using ten open-ended questions conceptually based in Baltes [4, 12, 13] life-span perspective was used to elicit information from a subset of 12 homeless veterans (from the total 39 participants) who agreed to participate in the study. The qualitative interview focused on questions regarding homeless veterans' expressed life experiences, military history, mental and physical health concerns, needs, and future goals. The interview questions are located in the appendix. 

### 2.8. Procedures

 The mixed-methods research effort aligned with Baltes' [4] life-span development theory with procedures indicative of both quantitative and qualitative research procedures. Veterans were provided with informed consent forms, with an explanation of the purpose of the study and the information they would be asked to provide, and they were informed of the option not to participate. Confidentiality of provided information was explained, and participants were asked to complete a brief demographic form, followed by a request to complete the Community Veteran Assessment. After completing the quantitative survey, participants were asked if they were interested in completing an interview with one of the researchers. Homeless veterans who volunteered to participate (*N* = 12) were interviewed in a separate area for privacy. Participants were provided information regarding available counseling services if needed while participating in the interview. Participants were also advised that they may discontinue participation at any time during the interview. Using the qualitative interview questionnaire, personal interviews of approximately 45–60 minutes each were conducted with the homeless veterans and service providers. Interview transcriptions were completed, representing more than 20 hours of personal interview discussions. 

### 2.9. Sample Description

 The sample for the study was comprised of 39 homeless veterans between the ages of 33 and 67 years. Seventeen homeless veterans from the Birmingham, Alabama, area and 22 homeless veterans from the Pensacola, Florida, area volunteered to complete surveys. The distribution of participants included approximately 64% Caucasian, 28% African-American, 5% Hispanic, and 3% Native American. Males outnumbered the females 5 : 1 with 33 males and six females. A complete demographic breakdown is presented in Table 1. 

### 2.10. Data Analysis

Data retrieved from the quantitative and qualitative instruments were analyzed relative to each of the seven research questions posited in the study. Each of the research questions is discussed relative to specific analysis of data procedures and study findings. 

## 3. Results and Discussion

Study findings are reported relative to each of the research hypotheses/questions and analysis of data discussions. 

### 3.1. Research Hypothesis  1

 There is a relationship between homeless veterans' levels of depression and the number of months they served in a war zone. 

Research hypothesis one was analyzed using the Pearson correlation procedure to determine if there was a relationship between the veterans' levels of depression and the number of months served in a war zone. The results indicated no statistically significant relationship with *r*(12) = .070, *P* > .05. Therefore, study findings did not support a relationship between homeless veterans' depression levels and the length of time they served in a war zone. 

### 3.2. Research Hypothesis  2

There is a relationship between service or no service in a war zone and whether or not the veteran becomes homeless. 

Data from research question two were analyzed using the nonparametric Chi Square analysis of data procedure to determine whether veterans' becoming homeless was related to whether or not they served in a war zone. Expected cell frequencies were examined to see whether there were any expected frequencies less than 5; the smallest expected frequency was 3.0. Therefore a Fisher exact test was performed, and these results indicated that there was no statistically significant relationship between whether or not veterans served in a war zone and their potential for becoming homeless: *χ* ^2^(1) = .650, *P* = .689. These results are also influenced by the small number of veteran participants in the study as demonstrated by the small cell frequencies resulting from the Chi Square Analysis findings. 

### 3.3. Research Hypothesis  3

There is a difference between homeless veterans and service providers in their median rankings of their perceptions concerning VA accessibility.

Research question three was analyzed using the Mann-Whitney *U* test to determine the difference between homeless veterans and service providers in their median rankings of perceptions concerning VA accessibility. The results of the Mann-Whitney test indicated that there was a statistically significant difference between the homeless veterans (Mdn = 3.0) and service providers (Mdn = 4.0), *U* = 258.50, *Z* = −2.06, *P* = .04, and *r* = .27. The median rank for providers was significantly higher than the median rank for homeless veterans, indicating that the VA accessibility was perceived to be positively accessible to service providers, while homeless veterans' perceptions of the VA reflected significantly less positive levels of accessibility. 

### 3.4. Research Hypothesis  4

There is a difference between the average perceptions of service providers in Northwest Florida and service providers in Birmingham, Alabama, regarding their perceptions of VA community partnership integration.

Research question four was analyzed using the Independent *t*-test procedure to determine mean differences in VA-Community partnership integration between the service providers from Birmingham, Alabama, and northwest Florida. The assumption of homogeneity of variance was assessed by the Levene test, *F* = 3.93, *P* = .064; this indicated no significant violation of the equal variance assumption; therefore, the pooled variances version of the *t*-test was used. The *t*-test for partnership integration indicated a significant difference in the means between Birmingham, Alabama, and Northwest Florida service providers with *t*(17) = − 2.64, *P* < .05, with Birmingham, Alabama, service providers M = 7.13, SD = 7.1 and northwest Florida service providers M = 8.73, SD = 8.7, on a scale of 0–10 (computed by the combined perceptions of VA accessibility and VA ability to coordinate services). The effect size, as indexed by *η* ^2^, was .05; this is a medium effect. The 95% CI for the difference between sample means, M_1_-M_2_, had a lower bound of −2.88 and an upper bound of −0.32. This outcome suggests a stronger partnership between the VA and community service providers in the northwest Florida region as compared with community service providers' partnerships with the VA within the Birmingham, Alabama, area.

### 3.5. Research Hypothesis  5

There is a difference in the rank ordering of the expressed needs of current study homeless veteran participants (from the Birmingham, Alabama, and northwest Florida regions) as compared with the national CHALENG 2010 study participants (from the Birmingham, Alabama, and northwest Florida regions). 

Research question five was analyzed by comparing homeless veteran participant expressed needs from each location (Birmingham, Alabama, and northwest Florida) to the corresponding locations in the CHALENG 2010 study. The Mann-Whitney *U* procedure was used to determine the difference in rank ordering of expressed needs of homeless veterans between current study participants and 2010 CHALENG study participants. There were no statistically significant differences in rank ordering of expressed needs of the current study homeless veterans from the Birmingham, Alabama, group when compared with the rank orderings of the CHALENG 2010 Birmingham, Alabama, group: *U* = 35.0, *Z* = 1.14, (*P* > .05). Similarly, a comparison of the current study homeless veterans participants' rank orderings of expressed needs from the northwest Florida group relative to the CHALENG 2010 northwest Florida group yielded no statistically significant differences in rank ordering, *U* = 50.0, *Z* = .00, (*P* > .05). A descriptive listing comparison of the top ten needs for each group is located in Table 2. 

### 3.6. Research Question  6

What are the major contributing factors to veteran homelessness conditions within Birmingham, Alabama, and northwest Florida? 

The qualitative analysis of data for research question 6 is integrated within the context and interpretation of research question 7. 

### 3.7. Research Question  7

What are the immediate needs of homeless veterans who are located within the Birmingham, Alabama, and northwest Florida regions? 

Analysis of data procedures for research questions 6 and 7 involved the retrieval of qualitative data from the *N* = 12 homeless veterans (a subset of the 39 homeless veteran study participants) who agreed to participate in the private interview sessions conducted by the researchers. Data gathered from interviews, recorded, transcribed, and then coded using thematic assessment, a data-driven research approach based on developing themes that answer a research question [17], were analyzed to respond to research questions six and seven. The analysis of data procedures included an integrated analysis and triangulation of data for generating inquiry-based explanatory results aligned with the constructs of a life-span development perspective. Interview data retrieved from the 12 study participants provided emergent themes with interpretive alignment to Baltes's life-span development model [4, 12, 13]. The emergent themes include lifelong or intermittent depression, drug use, and joining the service to escape current home life. The alignment between the theoretical model tenets of the life-span development perspective and the transitional perspectives of homeless veterans is reflected in selected participants' quotes in the following interpretive alignment discussions. Pseudonyms were used to protect the identity of the interviewees.

#### 3.7.1. Thematic Findings: Development Is Life-Long

The life-long tenet of the life-span development perspective posits that development does not peak or decline at a certain age and some elements of development occur across the life-span rather than in the first few years of life [4]. Life course does not decline with age; processes not present at birth emerge during the life-span in the form of problems, challenges, or adjustments to life. This life-long tenet was illustrated in the interview data as many homeless veterans explained their reasons for joining the military. The primary reason expressed by study participants for joining the military was to escape a dysfunctional or otherwise unhappy home life. Carl, a veteran who served between the Gulf and Iraq wars said: “I dropped out of high school and joined the Navy at age 17 to get away from an abusive father. Ray, who served 10 years in the Air Force reported, “I got my GED and joined the Army to get away from home.” A female veteran who joined the Army at age 22 (Rita) stated, “I was shooting for retirement, but [after 15 years] had to come out for health reasons.” Another veteran (Victor) whose life had been filled with problems of mental and physical illnesses and criminal charges stated, “I refuse to die until I reach happiness.”

#### 3.7.2. Thematic Findings: Development Is Sociocultural

According to the life-span development perspective [4], the setting and the time period of the individual's situation impacts his/her development. Sociocultural events can impact an individual's development in positive or negative ways, and for some individuals the inability to adapt may hinder the progress of development. Lifelong or intermittent depression was reported by all twelve of the homeless veterans who were interviewed. Depression may be a response to current life circumstances, environmental influences, or biological predispositions and may manifest immediately or as a delayed response [18]. Monty, a 31-year veteran of active Army and reservist service was recently shot in Afghanistan and forced to return home, but his grief has disrupted his ability to progress in every day civilian life: “I feel guilty leaving my buddies and I pray for them every night… I'm depressed more now than ever.” Cassie, a veteran of Desert Storm reported, “In a war zone, you do not have time to be depressed. After you're home for while… it just hits you.”

#### 3.7.3. Thematic Findings: Development Is Contextual

The life-span development perspective model emphasizes the contextual tenet [4]. Changing of individual resources occurs in response to needs in three areas: (1) growth, (2) maintenance or recovery, or (3) dealing with loss. Contextual changes can be in response to death, substance abuse, or mental illness with individual resources typically related to talent, time, money, energy, or social support. Drug use was another prevalent theme among the homeless veterans interviewed. Harold, a veteran of Vietnam reported, “I struggled with alcohol and drugs following my discharge from the army and felt in limbo… thinking about *woulda, coulda, shoulda* led to my drug use and I eventually just hit the road.” Rita reported she is recovering and “I haven't had a drink in over a month.”

#### 3.7.4. Thematic Findings: Development Is Multidimensional

The multidimensional tenet of the life-span development perspective [4] was exemplified in the interpretive interview data of study participants. Development cannot be described only in terms of increases or decreases in behavior but is comprised of multiple abilities and multiple pathways. Mitchell, an Army and Air Force veteran was a Licensed Practical Nurse (LPN) but lost his license because of a positive marijuana test. Although he cannot work as a nurse, he has used his social intelligence and life experiences to help other homeless veterans. “I do public speaking at different organizations to educate the community about homeless veterans. It is important for people to know that most people are just a couple of paychecks away from being homeless.” (Mitchell).

#### 3.7.5. Thematic Findings: Development Is Multidirectional

The multidirectional tenet of the life-span development perspective model [4] was demonstrated within participants' responses to interview questions. The development process consists of gains and losses, not necessarily in equal strength, and the balances between the two may change over time. Luke, a former Marine, reported “there is camaraderie between homeless veterans, regardless of branch of service and we talk about our issues—anger and anxiety—in a conversational environment, not in formal groups.” He also stated that the support of his friends assists him in his recovery from drug abuse and depression and “I'm intelligent and I want to go back to college—I want a job I can be proud of and deserve.”

#### 3.7.6. Thematic Findings: Development Is Plastic

The plasticity tenet of the life-span development perspective model [4] was revealed in the qualitative data analyses. Plasticity is the within-person variability potential to adapt to life's changes in order to maintain a positive outlook. After a life of military service, the deaths of family members, depression, substance abuse, and homelessness, Harold focuses on the positive aspects of his current life, “I'm currently doing better than I have in 15 years… I am grateful for the military, God, and country.” Mark acknowledged, “I'm the only one that can change my life.” Mario also reported a desire to “feel better both physically and psychologically.”

#### 3.7.7. Thematic Findings: Development Is Influenced by Biology and Culture

The biology and culture tenet of Baltes's [4] model was exemplified in the interpretive alignment to the theory. Biological potential weakens with age as an individual's goals and/or cultural support may increase. Victor, who suffers from numerous physical ailments and is currently under VA medical care reported, “I worked as a welder and landscaper but can no longer do such physically demanding jobs, I need vocational training so I can get a job.” Russell, a homeless veteran with a degree in Business Administration, was not coping as well as some of his peers and reported “I lost my job when the company I worked for closed. I just had surgery and the VA doctor is giving me antidepressants for depression, I cannot find a job and I cannot work construction or do any heavy lifting.” Russell's weakened physical state and the cultural change in his employment status as a result of the declining economy have influenced his development leading to a loss of functioning.

## 4. Conclusions and Implications

Capturing empirical and inquiry-based results that substantiate the life-span perspective relative to homeless aging veterans was supported by the study. Interview data, survey data, and triangulation analysis of data with interpretation provided evidence of major influences depicting the tenets and characteristics of Baltes [4] theory of life-span development perspective. Homelessness, war, deployment, mental health issues, experiences prior to entering the military, drug and alcohol use, childhood and adolescent experiences, dysfunctional relationships, and other multidimensional and multidirectional factors were evidenced in the empirical and qualitative data findings. Quantitative and qualitative study findings align with the seven tenets of Baltes' theory of life-span development perspective and highlight several of the constructs (plasticity, context, maintenance, and regulation of loss) as key contributors to explanatory factors influencing the life-span development of homeless aging veterans.

The scholarly significance of the study is highlighted within three contexts: (1) the role of the current study within the context of sensitive populations for conducting research; (2) the practical need for the current study within the context of prevention efforts aimed at homelessness; and (3) the connection of the theory of life-span development with the population focus of aging homeless veterans as the framework for conducting research within the context of adding a robust perspective for adding to the body of research aimed at poverty, homelessness, and aging veterans. 

 Rosenheck et al. [19] support specific lessons for researchers aimed at special populations of homeless Americans; that is, (a) the diversity needs of these special populations must be “identified, respected, and addressed” (page 1); (b) the uniqueness of the specific subgroup needs (aging homeless veterans) must be “sensitively addressed” (page 1); (c) systematic assessments must be included in the effort with specific subgroup populations; (d) the basic needs for the subgroup must include “tangible needs for material resources” (page 1); and (e) the need to increase the “overall pool of resources” for special population subgroups (page 1). The current study amplifies the types of considerations scholars and researchers must address for the special population and subgroups within aging homeless veterans and highlights the diversity, uniqueness, assessment, and needs for resources of aging homeless veterans as a research population addressed with respect and sensitivity. 

 The practical major need for prevention efforts to end veteran homelessness moves beyond the scholarly significance for the study; that is, the need for research must address strategies that focus on the health and economic challenges of veterans before they are threatened with the possibility of homelessness. The Department of Veterans Affairs initiated the Veterans Homelessness Prevention Demonstration (VHPD) Program in five cities in the spring of 2011 and has targeted those veterans who have completed assignments in the Middleeast [20]. Thus, although prevention and immediate intervention are the current focus of the VA for veterans returning from service to prevent or intervene prior to homelessness occurrences, the need for programs to serve aging homeless veterans currently representing almost half of the veterans who are homeless and who will not be included in the targeted Veterans Homeless Prevention Demonstration Program is the focus of this research effort and aligns with the significance of the need for this timely study. 

 Connecting the application of research associated with homeless aging veterans to life-span development theory provides a strong venue for scholars interested in examining the constructs of the life-span development perspective. Aligning empirical and qualitative data with appropriate triangulation interpretive information to the seven tenets of Baltes life-span development perspective [4, 12, 13] provides researchers and scholars sound information for adding to the body of literature on poverty, aging, and homelessness. 

President Obama's mandate to end veteran homelessness by 2015 is a strong declaration to the veterans who have served in the military and the providers who service them. In 2008 there were 154,000 homeless veterans and that number has been reduced to 59,000 in 2012 (Department of Veterans Affairs; National Coalition for Homeless Veterans). Although tremendous reduction in the number of homeless veterans has occurred since 2008, there remains much room for progress to reaching the 2015 goal. Findings of this study helped to frame the focus of research on homeless veteran experiences, specific characteristics, and needs, and how these factors influence life-span development. Understanding the contributing factors and how this unique population differs from nonveterans who are homeless is necessary to accomplishing this goal. In addition, service providers and the VA working cooperatively to establish programs aimed at assisting homeless veterans who may not have a physical, mental, or substance abuse diagnosis are recommended as an emerging need for future programs aimed at ending homelessness for veterans. Aging homeless veterans and female homeless veterans are two populations that have not been specifically addressed in the literature yet with the Vietnam generation nearing their mid-60s and increasing numbers of women serving in the military, these populations are among the most vulnerable to homelessness. Litz [21] suggested “the wars of Afghanistan and Iraq are the most sustained combat operations since the Vietnam war” (page 1). As these conflicts come to an end, a new generation of veterans will return home to a weakened economy to face joblessness and medical and mental health problems as well as possible homelessness. 

 Considering homeless veterans from a life-span development perspective may help service providers understand how sociocultural, contextual, and biological factors impact a veteran's development and adaptation to the changes from a structured military environment to life as a civilian. Each veteran can also contribute to helping fellow veterans as “occupational experts” with multidimensional knowledge. The issue of homeless veterans is not just a matter of finding homes for those who currently lack housing, but also establishing proactive programs aimed at preventing homelessness for those most at risk. 

## Figures and Tables

**Table 1:** Descriptive information for gender and ethnicity of homeless veterans by location (*N* = 39).

	Birmingham, Alabama	Northwest Florida
Gender	*n* = 17	*n* = 22
Males (*n* = 33)	14	20
Female (*n* = 6)	3	2
Ethnicity		
Hispanic (*n* = 2)	1	1
African-American (*n* = 11)	4	7
Native-American (*n* = 1)	1	0
Caucasian (*n* = 25)	11	14

**Table 2:** Top ten highest unmet needs identified by homeless veterans in 2010 and 2012.

	Birmingham, Alabama		Northwest Florida	
	2010 CHALENG project (*n* = 59)	2012 Current study (*n* = 17)	2010 CHALENG project (*n* = 120)	2012 Current study (*n* = 22)
1	Welfare payments	Dental care	Child care	Guardianship (financial)
2	Women's health care	Welfare payments	Legal assistance for child support issues	Long term/permanent housing
3	Guardianship (financial)	VA disability/pension	Family reconciliation assistance	Help with finding employment
4	VA disability-pension	Guardianship (financial)	Legal assistance to help restore driver's license	Child care
5	Family reconciliation assistance	Help managing money	Welfare payments	Help managing money
6	Legal assistance for outstanding warrants/fines	Credit counseling	Legal assistance for outstanding warrants/Fines	Family reconciliation assistance
7	Dental care	Job training	Dental care	Welfare payments
8	Legal assistance for child support issues	Legal assistance for outstanding warrants/fines	Reentry services for incarcerated veterans	Dental care
9	Help with finding employment	Family reconciliation assistance	SSI/SSD process	SSI/SSD process
10	Child care	Legal assistance for child support issues	Guardianship (financial)	Job training

## Appendix

### Qualitative Interview Questions

1. Tell me about your life story (any issues prior to service).
2. What was your reason for joining the military?
3. What can you tell me about the type of homecoming reception you received following your military discharge?
4. What type of support system do you currently have?
5. What is the biggest contributing factor to your current living situation?
6. What are your immediate needs?
7. What are your feelings regarding your military service and current circumstances?
8. How have things changed for you since you became involved with Three Hots and a Cot?
9. What do you want others to know about you and other veterans?
10. What is most important to you?
